# *SuMoToRI*, an Ecophysiological Model to Predict Growth and Sulfur Allocation and Partitioning in Oilseed Rape (*Brassica napus* L.) Until the Onset of Pod Formation

**DOI:** 10.3389/fpls.2015.00993

**Published:** 2015-11-17

**Authors:** Sophie Brunel-Muguet, Alain Mollier, François Kauffmann, Jean-Christophe Avice, Damien Goudier, Emmanuelle Sénécal, Philippe Etienne

**Affiliations:** ^1^INRA, UMR 950 Ecophysiologie Végétale, Agronomie et Nutritions N.C.S.Caen, France; ^2^Normandie UniversitéCaen, France; ^3^UNICAEN, UMR 950 Ecophysiologie Végétale, Agronomie et Nutritions N.C.S.Caen, France; ^4^INRA, UMR 1391 ISPAVillenave d’Ornon, France; ^5^Bordeaux Sciences Agro, UMR 1391 ISPAGradignan, France; ^6^UMR CNRS-UCBN 6139 Laboratoire de Mathématiques Nicolas Oresme, UFR des Sciences, Campus 2, Université de Caen Basse-NormandieCaen, France

**Keywords:** oilseed rape, sulfur, sulfate, temperature, photosynthetically active radiation, model

## Abstract

Sulfur (S) nutrition in rapeseed (*Brassica napus* L.) is a major concern for this high S-demanding crop, especially in the context of soil S oligotrophy. Therefore, predicting plant growth, S plant allocation (between the plant’s compartments) and S pool partitioning (repartition of the mobile-S *vs*. non-mobile-S fractions) until the onset of reproductive phase could help in the diagnosis of S deficiencies during the early stages. For this purpose, a process-based model, *SuMoToRI (Sulfur Model Toward Rapeseed Improvement*), was developed up to the onset of pod formation. The key features rely on (i) the determination of the S requirements used for growth (structural and metabolic functions) through critical S dilution curves and (ii) the estimation of a mobile pool of S that is regenerated by daily S uptake and remobilization from senescing leaves. This study describes the functioning of the model and presents the model’s calibration and evaluation. *SuMoToRI* was calibrated and evaluated with independent datasets from greenhouse experiments under contrasting S supply conditions. It is run with a small number of parameters with generic values, except in the case of the radiation use efficiency, which was shown to be modulated by S supply. The model gave satisfying predictions of the dynamics of growth, S allocation between compartments and S partitioning, such as the mobile-S fraction in the leaves, which is an indicator of the remobilization potential toward growing sinks. The mechanistic features of *SuMoToRI* provide a process-based framework that has enabled the description of the S remobilizing process in a species characterized by senescence during the vegetative phase. We believe that this model structure could be useful for modeling S dynamics in other arable crops that have similar senescence-related characteristics.

## Introduction

Sulfur (S) is an essential mesonutrient for optimal plant growth and development, ranking in need next to N, P, and K. It is required for the synthesis of essential S-containing amino acids (AAs; cysteine and methionine), S-derived compounds involved in key defense mechanisms and in other essential metabolic functions (e.g., co-factors, vitamins, enzymes, precursors for the synthesis of hormones) (Kopriva and Koprivova, 2005). During the last four decades, S nutrition has become a focus of attention due to observations of increasing areas of S deficient crops in Europe (Schnug and Evans, 1992; Scherer, 2001; McGrath et al., 2002). These outcomes are the consequence of (i) 1980’s legislation aimed at decreasing the level of sulfur dioxide from industrial emissions, which in due course drastically reduced these “free” S fertilizer depositions (Schnug and Evans, 1992) and (ii) the substitution of S-containing fertilizers and fungicides with alternative compounds containing no or low amounts of S (Zhao et al., 1999).

Rapeseed (*Brassica napus* L.) is a high S demanding crop because its needs are up to four times those of wheat (Postma et al., 1999; Oenema and Postma, 2003; Hawkesford and De Kok, 2006). Recent surveys have shown that up to 40% of rapeseed yield reduction has been attributed to S limitation. Negative impacts of S limitation have manifested as pod abortions (Postma et al., 1999) and weaker resistance to biotic and abiotic stresses (Bloem et al., 2004; Rausch and Wachter, 2005), as well as reductions in grain nutritional quality and more precisely lipid content, fatty acid composition (Ahmad and Abdin, 2000; Dubousset et al., 2010; D’Hooghe et al., 2014) and seed storage proteins, which are of interest for the meal that results from oil-extraction processes (D’Hooghe et al., 2014). The most drastic effects of S limitation on yield components and grain quality have been observed when they manifest at early growth stages (Dubousset, 2009; Dubousset et al., 2009, 2010). Other studies have reported that significant reductions in the leaf area (LA) expansion rate are observed when S restriction is applied from the beginning of the formation of side shoots, thus indicating that early S availability to the plant is a determinant of further growth (Haneklaus et al., 2008). During the vegetative phase, leaves are the most S-enriched compartments (up to 90% of the whole plant S; Dubousset, 2009). Between 30 and 60% of the total S in the leaves is in the form of sulfates (S-SO_4_^2-^), which makes the leaves the main sulfate storage compartment (Dubousset et al., 2009; Girondé et al., 2014). On top of that, during sequential senescence, leaves can fall with high residual S and S-SO_4_^2-^ contents during the vegetative phase (up to 3% and 1% of leaf dry matter for S and S-SO_4_^2-^, respectively) leading to substantial losses of S-SO_4_^2-^. Therefore, the balance in the leaves between the non-mobile-S fraction and the mobile-S fraction (mainly sulfates) that is readily available for remobilization toward growing organs indicates the potential to satisfy further S requirements in restricting S conditions and hence the plant S status. Therefore, predicting plant growth, S plant allocation (between the plant’s compartments) and S pool partitioning (repartition of the mobile-S *vs*. non-mobile-S fractions) until the onset of pod formation could help in the diagnosis of S deficiencies during the early stages.

Modeling approaches for rapeseed growth under major environmental constraints, i.e., mainly temperature and photosynthetically active radiation (PAR), have been undertaken previously (Diepenbrock, 2000; Jullien et al., 2011). They have generally aimed to predict biomass and yield and were derived from basic models originally developed for other crops. To date, the rapeseed models that have integrated the effects of mineral nutrition and their interactions with temperature and PAR have been mainly focused on N (Petersen et al., 1995; Habekotté, 1997a,b; Gabrielle et al., 1998a,b; Jeuffroy et al., 2003, 2006; Malagoli et al., 2004). In most of them, the formalisms related to N flux functioning take into account the process of senescence as a main contributor of remobilized N compounds and the establishment of the critical N dilution curve (Justes et al., 1994) as calibrated for rapeseed by Colnenne et al. (1998). However, while N limitation promotes leaf senescence and the induction of N remobilization due to increased proteolysis (Avice and Etienne, 2014), S limitation is neither a triggering nor an accelerating factor of leaf senescence because the main remobilizing form is sulfate, which is mainly remobilized from mature leaves (Dubousset et al., 2009).

These have several consequences for modeling plant growth, S allocation within plant compartments and S pool partitioning (mobile-S vs. non-mobile-S fractions). First, the senescence-related formalism is not dependent on the plant S status. Secondly, the estimation of the S-mobile pool shared by all organs, which is a concept that was already developed in other crop models (for N in wheat, Bertheloot et al., 2011a,b), is approximated by the quantification of sulfate. Thirdly, the critical S requirements for growth (as defined for N in Ulrich, 1952) match the S amount that is assimilated into organic forms (used for metabolic and structural functions) and not stored in the main mineral form (sulfate). As a consequence, the construction of the S dilution curve will consider S from the organic S forms solely, which will be approximated as the difference between S from all S-containing compounds and S from sulfates.

The objectives of modeling plant growth, S allocation between compartments and S pool partitioning are to provide a mechanistic framework to analyze the S remobilizing fluxes through the dynamics of the S mobile pool. Because this mobile pool in the leaves is an indicator of the S amounts that could be remobilized, its prediction would help in quantifying the potential to satisfy the S growth requirements toward growing sinks at the onset of pod formation. This paper describes *SuMoToRI* (*Sulfur Model Toward Rapeseed Improvement*), and its calibration and evaluation. We will explore the model’s behavior under contrasting S supply conditions with simulation tests performed with the dataset used for calibration of the model, and then we will evaluate the model with another independent dataset.

## Materials and Methods

### Model Description

#### Core Principles

**FIGURE 1:**
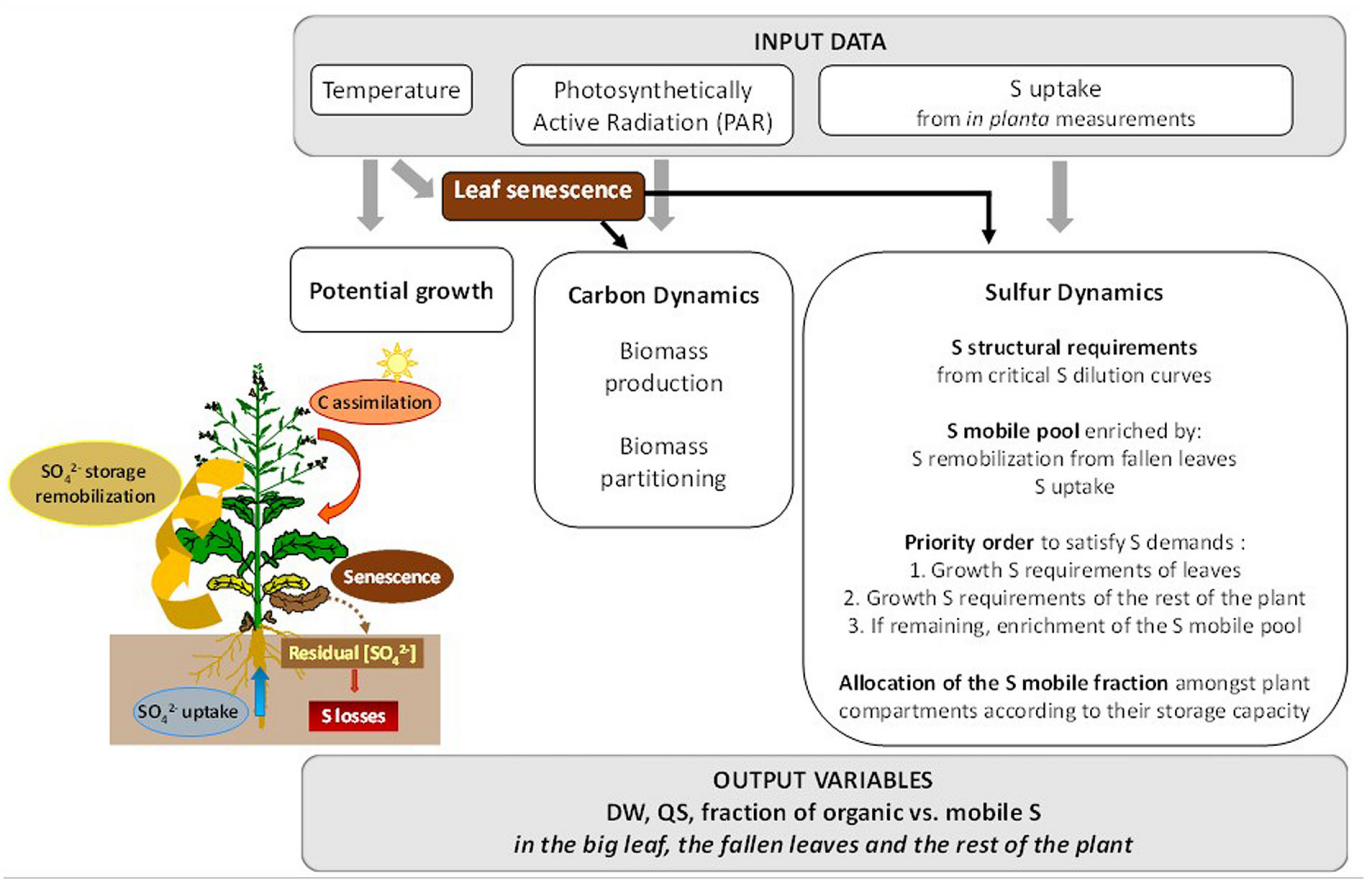
**Overview of *SuMoToRI.*** The model uses the following environmental data as inputs: temperature, photosynthetically active radiation (PAR) and S uptake (time courses adjusted from *in planta* measurements). These environmental factors drive (i) potential growth, (ii) the dynamics of C and (iii) the dynamics of S amongst the plant compartments. The main processes and underlying rules that are integrated in the model are indicated in the boxes: biomass production and partitioning for C dynamics and S allocation for growth requirements, i.e., structural and metabolic requirements (given by critical S dilution curves), which satisfy the leaves first and then the rest of the plant; and the composition of the S-mobile pool (derived from S uptake and remobilization from senescing leaves), which is allocated between the leaves and the rest of the plant according to allocation rules based on their capacity for SO_4_^2-^ storage. Leaf senescence dynamics impact carbon availability, the living tissues and sulfur availability as a consequence of the residual SO_4_^2-^ content in fallen leaves (FL), which represents a loss from the S-mobile pool shared by all organs in the plant.

*SuMoToRI* is a dynamic temperature-, PAR- and S-driven crop model (**Figure 1**). The predictive period encompasses the end of the vegetative rest period until pod formation. It is a compartment model that distinguishes *in planta* leaves simplified as a single big leaf (hereafter denoted as BL, including photosynthetic and senescing leaves), fallen leaves (FL) and the rest of the plant (hereafter denoted as rest, combining roots, taproot, stem, inflorescences and newly formed pods). Effective organ growth is calculated at each time step (1 day, which corresponds to a daily accumulated thermal time during day *i*, dTT*_i_*) from (i) potential organ growth as a function of temperature, (ii) carbohydrate availability hereafter denoted as carbon (C) offer (as generally developed in other crop models: DAISY-Rape, Petersen et al., 1995; CERES-Rape, Gabrielle et al., 1998a; STICS, Brisson et al., 1998; Azodyn-Rape, Jeuffroy et al., 2003, 2006; GRAAL-CN, Drouet and Pagès, 2003; Fussim-P- Maize, Mollier et al., 2008), and (iii) plant S offer which is defined as daily absorbed S plus the remobilized S pool.

The key variable of the model is the LA from which the other outputs are derived, i.e., total dry weight (TDW), dry weight of the big leaf and the FL (LDW_effective_ _BL_, LDW_FL_ respectively), S amounts in the big leaf and FL (QS_BL_, QS_FL_), amounts of mobile and organic S in leaves (QS_mobile_ _BL_, QS_org_ _BL_) and in the rest of the plant (QS_mobile rest_, QS_orgrest_). At each time step (dTT*_i_*) the effective increase in LA (dLA*_eff_*) is determined according to (i) thermal time, which gives the potential growth that only depends on temperature (dLA*_pot_*) (ii) absorbed PAR, which allows carbohydrate production and allocation to the leaves according to their demand with the driving rule that leaves are satisfied first (dLA*_carb_*) and (iii) S offer which is used to satisfy the growth requirements of the leaves first (dLA*_S_*). The daily effective increase in LA (dLA*_eff_*) is determined as the minimum of the three daily increases, i.e., dLA*_pot_*, dLA*_carb_*, and dLA*_S_*, assuming that one of these factors is limiting during dTT*i* and restrains the increase in LA (applying von Liebig’s “law of minimum”).

Except for PAR interception, all variables and processes are simulated at the plant level. The definitions of the variables and parameters are given in **Tables 1** and **2** respectively. Numbered equations in the following section are presented in the Appendices (**Supplemental Figure S1** and **Table S1**).

**Table 1:** Symbol, definition and unit of the variables used in the equations presented in the text and Appendices.

Symbol	Definition	Unit	Equations
dTTi	Increase in Thermal Time between day i-1 and day i	°Cd	Eq. 1, 5, 12
t	Time in days	d	
TTcum	Accumulated Thermal Time	°Cd	
**PAR interception**			
PARabs	Absorbed Photosynthetic Active Radiation above the canopy at day i	MJ m^-2^	Eq. 2
PAR_i_	Incoming photosynthetically active radiation above the canopy at day i	MJ m^-2^	
**Leaf area expansion**			
dLA_TT_	Potential increase in leaf area	m^2^ plant^-1^	Eq. 1
dLA_Carb_	Increase in leaf area according to carbohydrate production	m^2^ plant^-1^	Eq. 4
dLA_S_	Increase in leaf area according to S offer	m^2^ plant^-1^	Eq. 15
LA_BL_	Leaf area of the big leaf (BL)	m^2^ plant^-1^
LAI_BL_	Leaf area index of the big leaf (BL)	m^2^ m^-2^
LA_FL_	Leaf area of fallen leaves (FL)	m^2^ plant^-1^	
dLA_TOTeffective_	Effective increase in leaf area of all the produced leaves (BL and FL)	m^2^ plant^-1^	Eq. 18
**Biomass production**			
dLDW_TOTpot_	Potential increase in leaf dry weight of BL and FL	g DW plant^-1^	Eq. 3
LDW_TOTeffective_	Effective leaf dry weight of all the produced leaves	g DW plant^-1^	Eq. 18
LDW_effectiveBL_	Effective leaf dry weight of the big leaf	g DW plant^-1^	
LDW_FL_	Leaf dry weight of fallen leaves	g DW plant^-1^	
DW_rest_	Dry weight of the rest of the plant (stem, roots, taproot, pods)	g DW plant^-1^	
TDW	Total dry weight (including FL)	g DW plant^-1^	
**S amounts and contents**			
dQS_offer_	Increase in S offer	mg S plant^-1^	Eq. 10
dQS_uptake_	Increase S uptake	mg S plant^-1^	Eq. 5
dQS_mobile pool_	Increase in S-mobile pool	mg S plant^-1^	Eq. 13
QS_mobile pool_	Amount in the S-mobile pool (without S in FL)	mg S plant^-1^	
QS_FLpotremob_	Potential amount of remobilized S from FL	mg S plant^-1^	Eq. 11
dQS_FLmobile_	Increase in S-mobile pool in FL	mg S plant^-1^	Eq. 14
dQS_org_ _BL_	Increase in organic S in the big leaf (BL)	mg S plant^-1^	Eq. 6
dQS_orgrest_	Increase in organic S in the rest of the plant	mg S plant^-1^	Eq. 8
dQS_mobile_ _BL_	Increase in mobile S in the big leaf (BL)	mg S plant^-1^	Eq. 17
dQS_mobilerest_	Increase in mobile S in the rest of the plant	mg S plant^-1^	
QS_BL_	Amount of S in BL (including structural and mobile S)	mg S plant^-1^	
QS_rest_	Amount of S in the rest of the plant (including organic and mobile S)	mg S plant^-1^	
QS_TOT_	Total amount of S in the plant (excluding FL)	mg S plant^-1^	
QS_FL_	Amount of S in FL	mg S plant^-1^	
[S_BL_]_crit_	Critical S content in BL	mg S g^-1^ DW	Eq. 6, 7
[S_rest_]_crit_	Critical S content in the rest of the plant	mg S g^-1^ DW	Eq. 8, 9
[S_BL_]_org_	Content of organic S in BL	mg S g^-1^ DW	
[S_BL_]_mobile_	Content of mobile S in BL	mg S g^-1^ DW	

**Table 2:** Symbols, definitions and units of the parameters used in the equations presented in the Appendices.

Symbol	Definition	Unit	Equations
**Sowing condition**			
*ds*	Plant density	plant m^-2^	
**PAR interception**			
*k*	PAR extinction coefficient	m^2^ m^-2^	Eq. 2
**S uptake**			
QS_ini_	Initial S uptake	mg S plant^-1^	Eq. 5
aQS	Parameters of the function describing QS as a function of TT	mg S plant^-1^	
bQS		°Cd^-1^	
**Potential leaf growth**			
LA_0_	Initial leaf area of photosynthetic leaves	m^2^ plant^-1^	Eq. 1
LA_max_, K, *n*	Leaf area expansion parameters	m^2^ plant^-1^, °Cd, dimensionless	
**C acquisition and plant offer**			
PARabs_ini_	Initial absorbed PAR	MJ m^-2^	Eq. 2
TDW_ini_	Initial total dry weight	g DW plant^-1^	
RUE	Radiation use efficiency	g DW MJ^-1^	
DW_FLini_	Initial dry weight of fallen leaves	g DW plant^-1^	Eq. 12
aLDW_FL_	Parameters of the function describing the time progression of dry	g DW plant^-1^ °Cd^-1^	
bLDW_FL_	Weight of the fallen leaves	dimensionless	
**C allocation to leaves**			
β	Coefficient of dry weight allocation to the leaves	dimensionless	
**Big leaf C demand**			Eq. 3
LDW_BL_ _ini_	Initial dry weight of the big leaf	g DW plant^-1^	
SLA	Specific leaf area	m^2^ g DW^-1^	
**Growth S demand**			
α_BL_, β_BL_	Parameters to estimate critical S content in BL as a function of the dry weight of the BL	mg S plant^-1^ dimensionless	Eq. 7
α_rest_, β_rest_	Parameters to estimate critical S content in the rest of the plant as a function of dry weight of the rest of the plant	mg S plant^-1^ dimensionless	Eq. 9
**Mobile S allocation to leaves**			
ε_pot_	Coefficient of potential repartition of mobile S to the leaves	dimensionless	Eq. 17

#### Simulation of C Offer and Leaf C Demand

The plant carbon offer is provided by carbohydrate production from photosynthesis with the prior simplifications that C seed reserves are negligible at the stage of model initialization and C losses from respiration and photorespiration are not explicitly taken into account (as in other crop models, Yan et al., 2004; Mollier et al., 2008). The incoming PAR (PARi) is intercepted by the leaves and transformed into biomass in accordance with the radiation use efficiency (RUE) as described by Beer’s law and Monteith’s equation (Eq. 2; Gosse et al., 1983). Then, C allocation to the BL and the rest of the plant is defined according allocation rules based on sink strength. From an allometric relationship between leaf biomass and total biomass, a coefficient for biomass allocation to the leaves (β) is defined as the maximal leaf biomass to total biomass ratio. Accordingly, the daily increase in leaf area (dLA*_carb_*) is calculated from the fraction of biomass produced that is potentially allocated to leaves and the specific leaf area (SLA) (Eq. 4)

#### Establishment of Critical S Dilution Curves to Determine Plant S Requirements for Growth

It is assumed that the S requirements for growth represent the critical S amounts that are assimilated into organic compounds used for cell structures and metabolic functions, and not stored in the mobile pool.

For leaves, the critical S requirements are defined as the minimum amount of S accumulated in the leaves that yield the maximum leaf biomass (Ulrich, 1952). We assume that (i) critical S requirements are used to satisfy growth, i.e., structural and metabolic functions and as a consequence their estimation excludes any S storage forms, (ii) critical S requirements are satisfied by S-assimilated compounds, that is represented by S organic compounds, (iii) S storage forms are mineral compounds, mainly represented by sulfate (SO_4_^2-^) and (iv) the S organic fraction is the difference between the amount of total S and the amount of mineral S. Therefore, the daily increase in organic S-compounds in BL is driven by the daily critical S content in BL (Eq. 6), which is derived from a critical S dilution curve (Eq. 7) and the daily BL expansion. To build the critical S dilution curve, we used the method developed for N by Justes et al. (1994) and by Colnenne et al. (1998) for wheat and rapeseed, respectively. The storage form of S (S-SO_4_^2-^) was removed from the total S pool to consider only organic S compounds. Similarly, the daily increase in organic S-compounds for the rest of the plant (Eq. 8) was as a function of the critical S content of the rest of the plant (Eq. 9) and the daily growth of the rest of the plant.

#### Determination of Plant S Allocation within Plant Compartments

In the model, S offer is used to satisfy the requirements for growth (structural and metabolic functions) according to an order of priority, i.e., the growing leaves first and then the rest of the plant. Any remaining S amounts are stored (under the S form taken up by the plant, i.e., SO_4_^2-^) in a whole plant S-mobile pool that is shared by all organs and that can be used to satisfy the S growth demands of growing organs. The daily amount of S offer (Eq. 10) is the sum of (i) the daily S that is actually taken up by the plant and which is quantified from observed data (Eq. 5), (ii) the amount of remobilizable S from the FL (Eq. 11 and 12) and (iii) the pool of mobile S within the whole plant (Eq. 13).

To quantify the amount of remobilizable S from the FL, we relied on results showing that leaves are the richest compartment in S (and also in S-SO_4_^2-^) at these stages of development and that they fall with high residual amounts of S (mainly SO_4_^2-^). Therefore, the remobilization process is mainly driven by leaves that undergo senescence. The mobile form (S-SO_4_^2-^) in the FL before their fall (Eq. 11) is considered for the evaluation of the remobilizable S pool. The daily increase in leaf dry weight (LDW) of FL is based on an adjustment of the time courses, which is not dependent on plant S status (Eq. 12), as observed in Dubousset et al. (2009) and Abdallah et al. (2010).

Then, the variation in the pool of mobile S equals the daily uptake amount with the S amounts that were effectively remobilized from the FL and depleted of the S amounts required for growth (Eq. 13). The remaining S-SO_4_^2-^ in FL is calculated from the balance between the S requirements for growth and S uptake plus S remobilized from the mobile S pool (Eq. 14).

Then the daily increase in LA (dLA_S_) is calculated from the daily S offer, the SLA and the critical S content in the GL (Eq. 15).

#### Determination of the Mobile Fraction in the Leaves and in the Rest of the Plant

The mobile S fraction (S-SO_4_^2-^) allocated to the GL is determined by assuming that the pool of mobile S is allocated according to an optimal proportion based on the source size (i.e., BL). This proportion allocated to the BL is modulated by the level of S-SO_4_^2-^ remaining after satisfaction of the daily S requirements for growth. Therefore, if the daily S uptake is higher than the daily S requirements, the allocation is optimal otherwise the allocation coefficient is reduced according to observations under S restricting conditions (Eq. 16). Consequently, the fraction of mobile S in the rest of the plant is the remaining S-SO_4_^2-^ from the mobile pool (Eq. 17).

#### Determination of the Effective Growth

The effective increase in total LDW results from the daily effective increase in LA (Eq. 18). From this, the actual increase in LDW for BL is deduced by taking into account the daily leaf fall (Eq. 12).

### Experiments for Model Calibration and Evaluation

#### Plant Cultivation

Two experiments were performed from January until July in 2011 for Experiment 1 and in 2013 for Experiment 2. Data from Experiments 1 and 2 were used for model calibration and model evaluation, respectively. Plants of cv. Yudal were grown in a greenhouse at the UMR 950 EVA in Caen, France (49° 10′ N, 0° 21′ W) for 40 days (Experiment 1) and 38 days (Experiment 2) in the greenhouse under natural day/light conditions, and put into a climatic chamber maintained at 5°C for vernalization for 3 weeks (Experiment 1) and 4 weeks (Experiment 2) with a photoperiod of 16h (day) and 8h (night). Plants were then transferred into individual pots (mix of 2:3 perlite and 1:3 vermiculite) distributed randomly in the greenhouse at a plant density of 40 plants m^-2^ (Experiment 1) and 30 plants m^-2^ (Experiment 2), until the onset of pod formation.

Nutritional growing conditions were similar in Experiments 1 and 2. Plants were grown under two contrasting S supplies under non-limiting N availability. According to the relative-addition rate nutrient-dosing system (Ingestad and Lund, 1986; Oscarson et al., 1989), nutrient addition was increased over time to match plant growth requirements in order to produce a constant relative growth rate during exponential plant growth (**Supplemental Table S2**). The contrasting S treatments were determined as follows: the high S (HS) treatment supplied four times the calculated optimal requirements, and the low S (LS) treatment accounted for 5% of the optimal S requirements. Optimal S requirements were calculated from *in planta* measurements in non-limiting S plants based on data obtained from Dubousset et al. (2010). For the calculation of N requirements throughout the cycle, the time courses of shoot biomass of the same control plants (Dubousset et al., 2010) were used to calculate the amounts of daily N within the shoot for optimal plant growth by using the dilution curve established by Colnenne et al. (1998) in rapeseed and taking into account plant density. Daily amounts of S and N were provided by solutions of MgSO_4_ and NH_4_NO_3_ (**Supplemental Table S1**). During vernalization, plants of all the treatments were provided with a 25% Hoagland nutrient solution.

#### Phenological and Biochemical Measurements

Plants were harvested from the end of the vegetative rest period, which was 480°Cd (Experiment 1) and 565°Cd (Experiment 2) after sowing until the onset of pod formation (with a base temperature of 5°Cd, Morrison et al., 1992). Measurements were made at several representative growth stages (following the BBCH decimal system, Lancashire et al., 1991) from stem extension-six to the ten-leaf stage (GS16–GS20, end of vernalization), late budding (GS30), inflorescence emergence (GS50), early flowering (GS60) and early pod formation (GS70). Phenological measurements (LA and dry weights of *in planta* leaves, FL, and the rest of the plant) and biochemical analyses (amounts of S, N and SO_4_^2-^ in the different plant compartments) were performed as described in Brunel-Muguet et al. (2015). Measurements of SO_4_^2-^ were performed only for Experiment 1. Therefore, SO_4_^2-^ contents in Experiment 2 were estimated from the time courses of the S:SO_4_^2-^ ratio, which were determined for data from Experiment 1. The environmental variables necessary to run the model, i.e., daily PARi and maximum and minimum temperatures were recorded with line quantum sensors (LI-191, Eurosep Instruments) set at the top of the canopy and temperature probes (105T Campbell, Campbell Scientific Ltd., Leicestershire, UK).

### Model Implementation and Statistical Assessment of Performance

The model was implemented with the R language environment for statistical computing, version 2.9.1 (R Development Core Team, 2009). The predictive quality of the model was evaluated with the root mean square error (RMSE, in the same unit as the variable), which provides the mean difference between *n* predicted and observed values and, the index of agreement (*d*, Willmott, 1982), which indicates the degree of agreement between simulated values with their corresponding observed values. A low RMSE and a *d* value close to one unit are targeted when evaluating the performance of model simulations. The RMSE was calculated as follows:

$$RMSE=\sqrt{\sum_{i=1}^{n}{\left(S_{i}-O_{i}\right)}^{2}}/n$$

where *S_i_* and *O_i_* are the simulated and observed values at time *i*, and *n* is the number of observations. The index of agreement, *d*, was calculated using the following equation:

$$d=1-\frac{\sum_{i=1}^{n}{\left(S_{i}-O_{i}\right)}^{2}}{\sum_{i=1}^{n}\left[(S_{i}-O_{m}\right)+(O_{i}-O_{m}){]}^{2}}$$

where *O_m_* is the mean of the observed values.

### Model Calibration

Model calibration was performed with the dataset from Experiment 1. Parameter estimations were acquired by fitting to observations for each of the model’s functions from the end of vernalization (**Supplemental Figure S1** and **Table S1**). **Tables 3** and **4** give the calibrated parameter values and initialization states for both S conditions.

**Table 3:** Parameter values of *SuMoToRI* used for model calibration under HS and LS conditions (with dataset from Experiment 1).

Symbol	Definition	HS	LS	Unit	Source
PAR interception	
k	PAR extinction coefficient	*k* = 0.75		m^2^ m^-2^	Bonhomme et al., 1982
**Potential leaf growth**	
LA_max_	Leaf area expansion parameters	LA_max_ = 0.20		m^2^ plant^-1^	Estimated
K		*k* = 872.96		°Cd^-1^	
N		*n* = 6.31		dimensionless	
**C acquisition and plant offer**	
RUE	Radiation use efficiency	4.59	3.11	g DW MJ^-1^	Estimated
aLDW_FL_	Parameters of the function describing the time	0.0092		g DW plant^-1^ °Cd^-1^	Estimated
bLDW_FL_	progression of LDW_FL_	0.0043		dimensionless	
**C allocation to leaves**	
β	Coefficient of DW allocation to the leaves	0.41		dimensionless	Estimated
**C demand of the big leaf**	
SLA	Specific leaf area	0.028		m^2^ g DW^-1^	Estimated
**Growth S Demand**	
α_BL_	Parameters to estimate critical S content in BL as a	5.11		mg S plant^-1^	Estimated
β_BL_	function of LDW_BL_	-0.52		dimensionless	
		For LS: threshold value [S]_BL_crit = 3 mg S g DW^-1^ for LDW_BL_ < 3 g plant^-1^			
α_rest_	Parameters to estimate critical S content in the rest of the plant as a function of DW_rest_	1.83		mg S plant^-1^	Estimated
β_rest_		-0.004		dimensionless	
**Potential mobile S allocation to leaves**	
ε_pot_	Coefficient of potential repartition of mobile S to the leaves	0.8		Dimensionless	Estimated

**Table 4:** Initial state values under HS and LS conditions for model calibration (Experiment 1) and evaluation (Experiment 2).

Symbol	HS-Experiment 1	HS-Experiment 2	LS-Experiment 1	LS-Experiment 2	Unit
**Potential leaf growth**			
LA_0_	0.016	0.014	0.013	0.014	m^2^ plant^-1^
**C acquisition and plant offer**					
PARabs_ini_	0	0	0	0	MJ m^-2^
TDW_ini_	0.652	1.031	0.428	0.778	g DW plant^-1^
DW_FLini_	0	0.05	0	0.04	g DW plant^-1^
**C demand of the big leaf**					
LDW_BL_ _ini_	0.510	0.736	0.328	0.589	g DW plant^-1^
**S uptake**					
QS_TOTini_	8.799	10.594	2.865	1.939	mg S plant^-1^
aQS	7.540	23.457	3.14	2.207	mg S plant^-1^
bQS	0.0033	0.0026	0.0021	0.0014	°Cd^-1^
QS_BLini_	7.48	7.89	2.40	1.38	mg S plant^-1^
QS_restini_	1.32	2.26	0.47	0.55	mg S plant^-1^

*aQS and *bQS* are the parameters of the S uptake function. Their values are adjusted for each experiment (input variables).*

**FIGURE 2:**
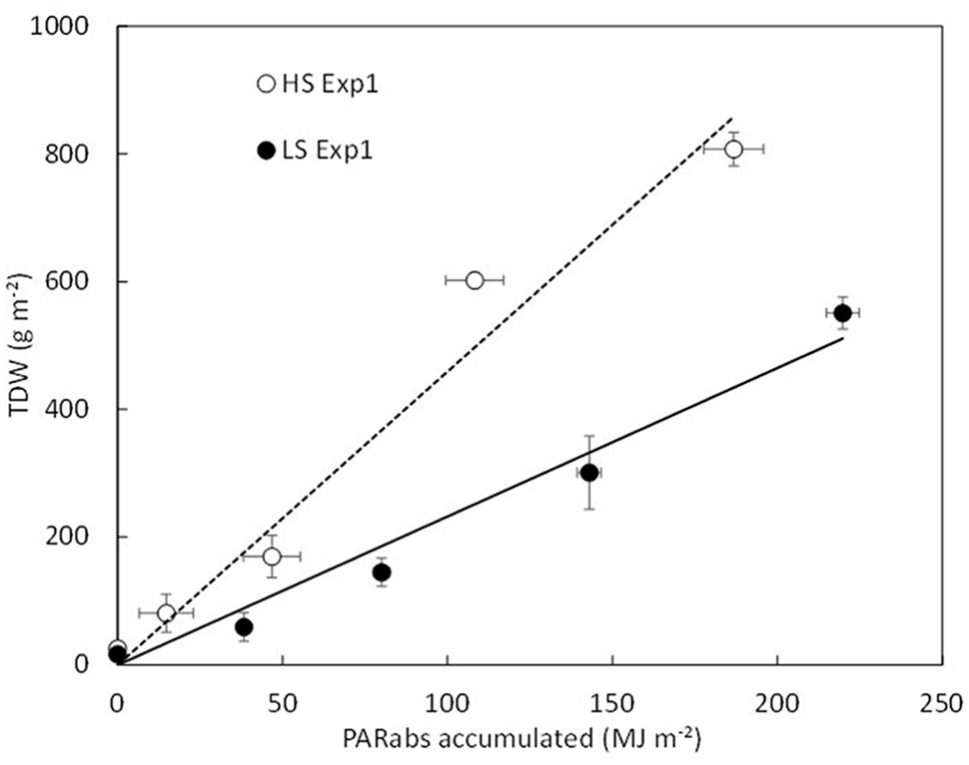
**Total dry weight (TDW) as a function of accumulated PAR_abs_ for HS (empty symbol, dotted line) and LS (filled symbol, full line).** There is a statistical difference in the slope of the relationship between HS (*RUE = 4.59 MJ.m^-2^)* and LS (*RUE = 3.11 MJ.m^-2^*), (RUE, *P < 0.05*). Bars denote standard deviations.

Adjusted parameters in HS and LS conditions were not significantly different except for the radiation use efficiencies (RUEs) (**Table 3**). Indeed, distinct RUE values were used according to the S condition. As illustrated in **Figure 2**, the RUE value that corresponds to the slope of the relationship TDW *vs*. accumulated PAR_abs_ was significantly lower in LS than in HS (*P < 0.05*). The critical S demand for BL cannot be applied to low biomass. Therefore, when LDW_BL_ < 3 g plant^-1^ (as observed in LS conditions), the critical S content in BL is assumed to be constant and is set at 0.3% LDW_BL_ (**Table 3**).

## Results

### Test of the Model’s Behavior

The model’s behavior was tested by comparing observations from Experiment 1 and simulations performed with the parameter values obtained from calibration of each process equation.

**FIGURE 3:**
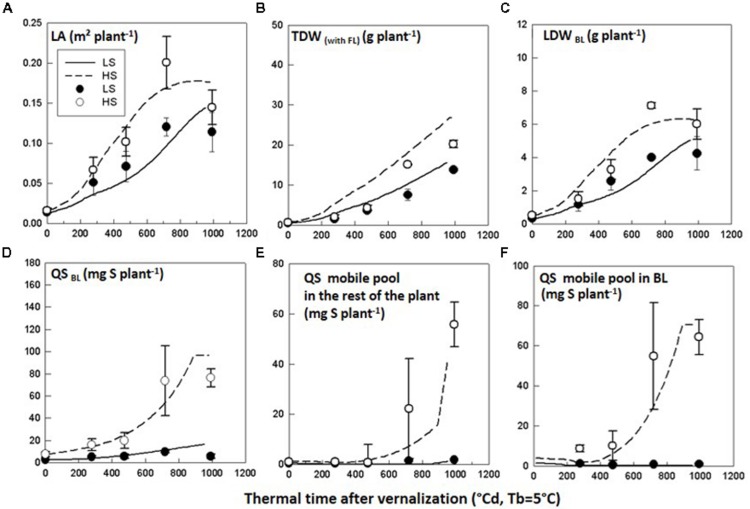
**Test of the model behavior.** Observed (symbols) and simulated (lines) time courses of **(A)** leaf area (LA), **(B)** total dry weight (TDW), **(C)** leaf dry weight of the big leaf (LDW_BL_), **(D)** total amount of S in the big leaf (QS_BL_), **(E)** amount of S-mobile pool in the rest of the plant (QS_mobile pool_ _rest_), and **(F)** amount of S-mobile pool in the big leaf (QS_mobile_ _BL_) for *Brassica napus* plants grown in LS and HS conditions in Experiment 1. The observed data are the means of three replicates. The vertical bars denote standard deviations.

Simulated LA, TDW and LDW_BL_ with *SuMoToRI* gave a good fit with the observed values with low RMSE values and *d*-values close to 1 (**Figures 3A–C**, **Table 5**). The difference in LA, TDW and LDW_BL_ between HS and LS was correctly simulated. The LA expansion rate increased until full flowering and started decreasing under both S conditions at the onset of pod formation (980°Cd). This was the consequence of the fall of senescent leaves, which was not compensated by the production of new leaves. The TDW increased under HS and LS, but while differences between S conditions were observed from 718°Cd, the simulated time courses became distinct slightly earlier than this (**Figure 3B**). Simulated QS_BL_ correctly indicated the significant difference between HS and LS with low RMSE values and high *d*-values (**Figure 3D**, **Table 5**). However, while simulations in HS nicely reproduced the plateau at full flowering, the QS_BL_ in LS was slightly overestimated at 980°Cd (**Figure 3D**, **Table 5**). The abrupt change in the QS_BL_ trend (at 900°Cd) under HS was due to the threshold condition used to estimate the daily value of mobile S in BL and was considered as null when the daily growth S requirements of BL turned negative. This negative value is the consequence of the negative daily value of LDW_BL_ because the production of dry matter for leaves no longer compensates for the biomass of FL (which was at its highest at this stage). The amounts of mobile S in BL and in the rest of the plant (**Figures 3E,F**) also correctly differentiated the two S-treatments. In the HS condition, the QS mobile pool in BL and in the rest of the plant were low until 450°Cd. It increased in both compartments in an exponential trend following the same pattern as the LA expansion rate. The RMSE values were low under both treatments but a lower model predictive quality was observed in the LS conditions with lower *d*-values in LS (**Table 5**). This might be due to the difficulties in calibrating the functions related to S pool partitioning (i.e., mobile fraction in the BL) because little material remained at full flowering for quantifying SO_4_^2-^ in the BL. However, consistent with our driving hypothesis, the amounts of SO_4_^2-^ representing the main mobile S form were close to zero under the most limiting S supply conditions (**Figures 3E,F**).

**Table 5:** Root mean square errors and *d*-values of the main variables for model calibration (Experiment 1) and model evaluation (Experiment 2) under HS and LS conditions.

	Experiment 1				Experiment 2			
	HS		LS		HS		LS	
	RMSE	*d*	RMSE	*d*	RMSE	*d*	RMSE	*d*
LAI	0.02	0.96	0.02	0.94	0.02	0.95	0.02	0.71
TDW	4.55	0.93	1.86	0.96	6.03	0.92	4.76	0.75
LDW_BL_	0.76	0.97	0.58	0.96	2.61	0.79	1.80	0.71
QS_BL_	12.9	0.95	5.0	0.52	21.9	0.92	1.1	0.44
QS mobile pool in BL	7.0	0.97	0.9	0.22	24.3	0.88	0.3	0.47
QS mobile rest	16.6	0.86	0.9	0.46	8.5	0.88	0.2	0.54

### Model Evaluation

**FIGURE 4:**
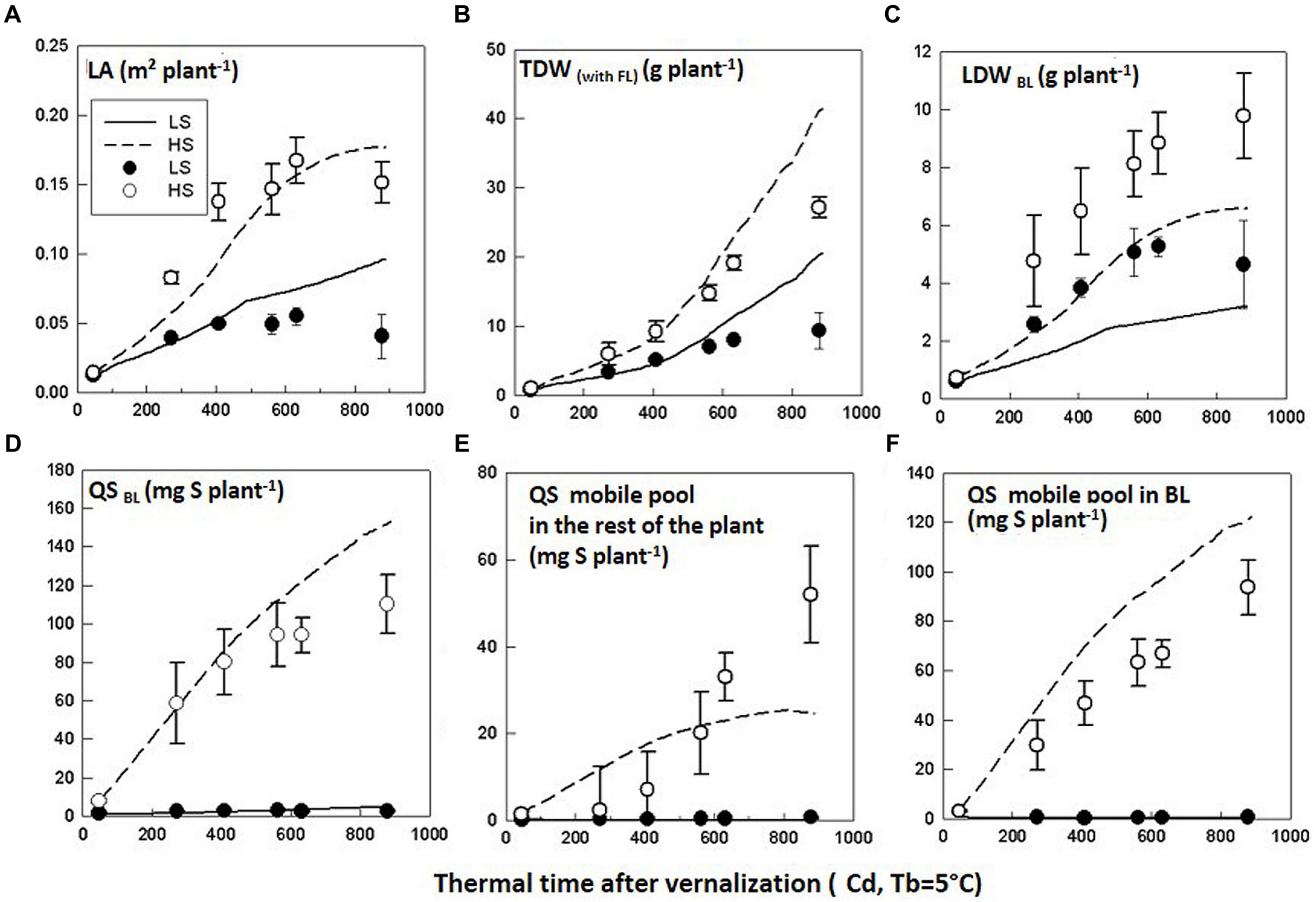
**Evaluation of the model.** Observed (symbols) and simulated (lines) time courses of **(A)** leaf area (LA), **(B)** total dry weight (TDW), **(C)** leaf dry weight of the big leaf (LDW_BL_), **(D)** total amount of S in the big leaf (QS_BL_), **(E)** amount of S-mobile pool in the rest of the plant (QS _mobile pool rest_), and **(F)** amount of S-mobile pool in the big leaf (QS_mobile_ _BL_) for *Brassica napus* plants grown in LS and HS conditions in Experiment 2. The observed data are the means of four replicates. The vertical bars denote standard deviations.

The model evaluation was performed by comparing observations from Experiment 2 and simulations performed with the parameter calibration values obtained from Experiment 1. Recorded daily PARi and thermal time and S uptake time courses from Experiment 2 (**Table 4**) were used as inputs for the simulations. In the HS condition, the simulations correctly predicted the time courses of LA, TDW and LDW_BL_, although TDW was overestimated and LDW_BL_ was underestimated especially from GS50 (**Figures 4A–C**, **Table 5**). This could be explained by the fact that the SLA value was assumed to be constant and was averaged throughout the simulation period, although it tended to slightly decrease (data not shown), meaning that the C demand of leaves increased. Therefore, the simulated LDW_BL_ with a decreased SLA should have been higher as observed. With regards to QS_BL_, QS_mobile pool_ _rest_, and QS_mobile_ _BL_, simulated time courses were correctly predicted in the HS and LS conditions but QS_BL_ and QS_mobile_ _BL_ were overestimated while QS_mobile pool rest_ was underestimated in the HS condition, especially at the end of the simulated period (**Figures 4D–F**, **Table 5**). This highlighted that the simulated repartition of the S-mobile form between the leaves and the rest of the plant (with the coefficient of potential repartition of mobile S to the BL and its conditions of modulation), was favorable for the leaves to the detriment of the rest of the plant. This could be accounted for by the low amount of the mobile form (SO_4_^2-^) in the BL of Experiment 2 which was estimated from the time courses of the S:SO_4_^2-^ ratio adjusted from the data of Experiment 1. In the LS condition, the model correctly simulated the trends, although simulations also underestimated LDW_BL_ (**Figure 4C**, **Table 5**). Simulated QS_mobile pool_ and QS_mobile_ _BL_ in LS were correctly predicted with low RMSE values and *d*-values that were even higher than the ones obtained through testing of the model behavior (**Table 5**). Globally, the values were consistent with the very low mobile fraction contents that were observed under LS.

## Discussion

### Accuracy of the Model Predictions and Genericity of the Parameter Values Under Contrasting S Supplies

The model parameters are generic, meaning that their values were applicable whatever S supply condition, with the exception of the RUE because the calibration of the function related to the conversion of PAR_abs_ into biomass was S-specific. The model accuracy was greatly improved with the S-specific calibration for RUE when testing and evaluating the model.

Recent studies have highlighted perturbations of C-related processes, i.e., photosynthesis, C metabolism and reactions related to oxidative stress in the young leaves of oilseed rape plants grown under S limitation (D’Hooghe et al., 2013). These authors reported that reduced photosynthesis was not associated with a decline in chlorophyll content, which remained stable, but rather with disturbances occurring during (i) the last steps of the photosynthetic electron transport chain in the thylakoid membrane (impairment in the reduction of NADP^+^ further required in the Calvin cycle) and (ii) the CO_2_ assimilation process (Jebanathirajah and Coleman, 1998; Maruyama-Nakashita et al., 2003). S restriction has also been shown to modify membrane composition with a decrease in sulfolipid content in the thylakoid membrane of the chloroplast (Nikiforova et al., 2003). This could impact the energy transport system and hence lead to impairments in the final electron transfer steps. This perturbation of the energy transport system could also be the consequence of alterations of the Fe–S centers that serve as early electron acceptors in photosystem I, under S restriction in *Arabidopsis* (Nikiforova et al., 2003). Simultaneously, evidence for oxidative stress was observed in young leaves of S-limited oilseed rape plants with the accumulation of H_2_O_2_ and anthocyanins acting as antioxidants (D’Hooghe et al., 2013). These authors also reported an increase in the abundance of key enzymes that catalyze the synthesis of jasmonic acid and ethylene, both inducing plant responses against biotic and abiotic stresses (Wi et al., 2010). The accumulation of H_2_O_2_ might be accounted for by the perturbations of enzymes under S limitation (amongst them glutathione-*S*-transferase), which are involved in the H_2_O_2_ detoxification process. Therefore, it seems that even though light reactions were affected by oxidative stress or globally maintained to provide stable energy production via reorientation of C metabolism (decrease in sucrose and starch synthesis and induction of glycolysis, Nikiforova et al., 2005), dark reactions were also rapidly repressed by S limitation leading to reduced CO_2_ assimilation (D’Hooghe et al., 2013). In relation to our observations of a lower RUE value under S restriction, this would mean that the efficiency of transforming energy from PAR into carbohydrates (dark reactions) is reduced and is not associated with the efficiency of capturing radiation (light reactions). Our results highlighted the importance of predicting S fluxes at the process level in order to provide mechanistic explanations and hypotheses that could be integrated in the model formalisms (Calderwood et al., 2014).

### Prediction of S Requirements for Growth

In the model we made two driving hypotheses regarding S requirements for growth (gathering structural and metabolic functions): (i) they are calculated as the difference between total S and S from mineral forms (ii) S mineral forms are mainly sulfate and as a consequence the S mobile pool takes into account sulfate only. However, this approximation of the mobile pool does not include other mobile forms that might enrich the mobile pool after degradation of s organic compounds under high S restrictive conditions. Indeed, glutathione and glucosinolates, which can account respectively for 2 and 6% of the total S in young leaves, were shown to be modulated by the intensity of S restriction (Blake-Kalff et al., 1998; Nikiforova et al., 2005). In addition, recent studies have highlighted the importance of methiin (*S*-methyl cysteine sulfoxide), which can account for up to 30% of the whole plant’s mobile AAs (Gaudin, 2013). Its presence in the phloem and in senescing leaves suggested that it had a major role in the N and S remobilization processes as observed in pea (Tan et al., 2010). Our observation and simulation results showed low amounts of the mobile pool of S in S-limiting conditions but this may not be an accurate estimate of the actual remobilizing pool of S compounds when S restriction is severe and where other mobile forms can be more important than sulfate. Therefore, as an improvement to the calibration of the model, the construction of the S critical dilution curve could take into account quantification of these S-compounds.

### Perspectives of Model Development

In the light of this work, two avenues for the future development of *SuMoToRI* can be foreseen.

First, in the present version, *SuMoToRI* does not include predicted S uptake from the soil. Other rapeseed models have developed a soil module to quantify N fluxes transferred from soil to the roots based on (i) the balance-sheet method (Rémy and Hébert, 1977; Machet et al., 1990) to predict net N soil mineralization combined with a prediction of nitrate leaching (Burns, 1976), N from FL (Dejoux, 1999) and a coefficient of effective uptake as in Azodyn-Rape (Jeuffroy et al., 2003) or (ii) the functioning of nitrate transporters and root sizes as in CERES-Rape (Gabrielle et al., 1998a) and in Malagoli et al. (2004). These approaches should be further investigated to consider the S mineralization processes in the soil (Suhardi, 1992) and the features of sulfate transporters (Takahashi et al., 2000).

Secondly, the period of prediction could be extended by taking into account all the photosynthetic surfaces after pollination, thus including autotrophic pods (Leterme, 1985) and this would substitute the current central variable, i.e., the LA index, for the green area index as a sum of LA and pod area indices (Jeuffroy et al., 2006). The prediction of S content in the pods at maturity is crucial because seeds of rapeseed are also a source of S-containing proteins, which is, amongst others, a quality criterion for cattle cakes. To further predict the content of S-containing proteins from the seed S content, the amounts of N-compounds used for protein synthesis must be also estimated (Martinez et al., 1987). This requires consideration of the effects of N availability on the S-related processes that are included in the model. Consistent with this requirement, it has been long established that there is a regulatory crosstalk between S, N and C metabolism that controls S assimilation (Reuveny et al., 1980; Zhao et al., 1997; Kopriva and Rennenberg, 2004). In addition, because leaf senescence is a key mechanism that controls the availability of the mobile S amounts allocated to growing pods and because it is tightly controlled by N availability (Dubousset et al., 2010; Abdallah et al., 2011), estimation of N compounds in the leaves at the onset of pod formation is necessary to further predict S-containing proteins as an indicator of grain quality.

Finally, the current structure of this process-based model offers a generic framework for modeling S dynamics in other crop species with similar senescence-related characteristics.

## Conclusion

Modeling the vegetative growth in rapeseed under S restriction is of major importance to predict crop responses and the S remobilization potential for growing sinks during this highly S-sensitive period. To reach these objectives, the process-based model, *SuMoToRI*, was developed with novel features relative to other mineral-driven crop models. It is based on two key principles: (i) the concept of N dilution generally shared by crop modelers, which has been adapted to estimate the S requirements for growth via the functioning of S fluxes and (ii) the quantification of an S-mobile pool used to satisfy the demand for growth (gathering structural and metabolic functions) and to indicate the remobilization potential of newly formed sinks. It is run with a small number of parameters with generic values, except in the case of RUE, which was shown to be modulated by S supply. Model simulations gave reliable predictions for the dynamics of LA, dry weights, S allocation between the compartments and S partitioning such as fraction of S-mobile compounds in the leaves, which is an indicator of the S remobilization potential. The mechanistic features of *SuMoToRI* provide a process-based framework that has enabled the description of the S remobilizing process in a species characterized by senescence during the vegetative phase. We believe that this model structure could be useful for modeling S dynamics of other arable crops with similar senescence-related characteristics.

## Author Contributions

SB-M, ES, DG, and J-CA contributed to the experimental design and tissue sampling. SB-M, J-CA, PE, and AM established the ecophysiological hypotheses to build the model. AM and SB-M constructed the model. FK wrote the model with the R language. SB-M performed the whole raw data analysis. SB-M and AM interpreted the data and wrote the article. J-CA, PE, and FK were involved in revising the manuscript for important intellectual content.

## Conflict of Interest Statement

The authors declare that the research was conducted in the absence of any commercial or financial relationships that could be construed as a potential conflict of interest.

## Supplementary Material

Figure S1 **Workflow of *SuMoTORI*.** The model predicts leaf area expansion, biomass and the S amounts in the whole-plant and in the plant compartments and the fraction of organic S (used for growth, i.e., structural and metabolic functions) vs. mobile S (estimated by S from SO_4_^2-^), for the big leaf, the FL and the rest of the plant (roots, taproot, stem, inflorescences, and pods) from the end of vernalization up to the onset of pod formation. Arrows indicate the precedent and dependent variables. Flat-ended lines represent subtraction of the variable (for details, see the related equation). Numbers in circles indicate the priority order to satisfy S demands from the S offer. State variables are framed with dotted lines. Definitions of the abbreviated variables and parameters are given in **Tables 1** and **2**. Equations are given in **Supplemental Tables S1**.

TABLE S1 **List of equations**.

TABLE S2 **Weekly (W) amounts of MgSO4 (mg) and NH4NO3 (mg) per plant for HS and LS treatments.** Between W5 and W6, there were 3 weeks of vernalization (Exp1) and 4 weeks of vernalization (Exp2) during which plants of all treatments were provided with a 25% Hoagland nutrient solution (see Material and Methods section). Amounts were calculated from sowing until seed maturity to match physiological stage S-requirements as observed in Dubousset et al. (2010).
