# Supplementary figures and images for: SuMoToRI, an Ecophysiological Model to Predict Growth and Sulfur Allocation and Partitioning in Oilseed Rape (Brassica napus L.) Until the Onset of Pod Formation
Source: Front Plant Sci. 2015 Nov 17;6:993. doi: 10.3389/fpls.2015.00993 (PMC4647072; doi:10.3389/fpls.2015.00993)

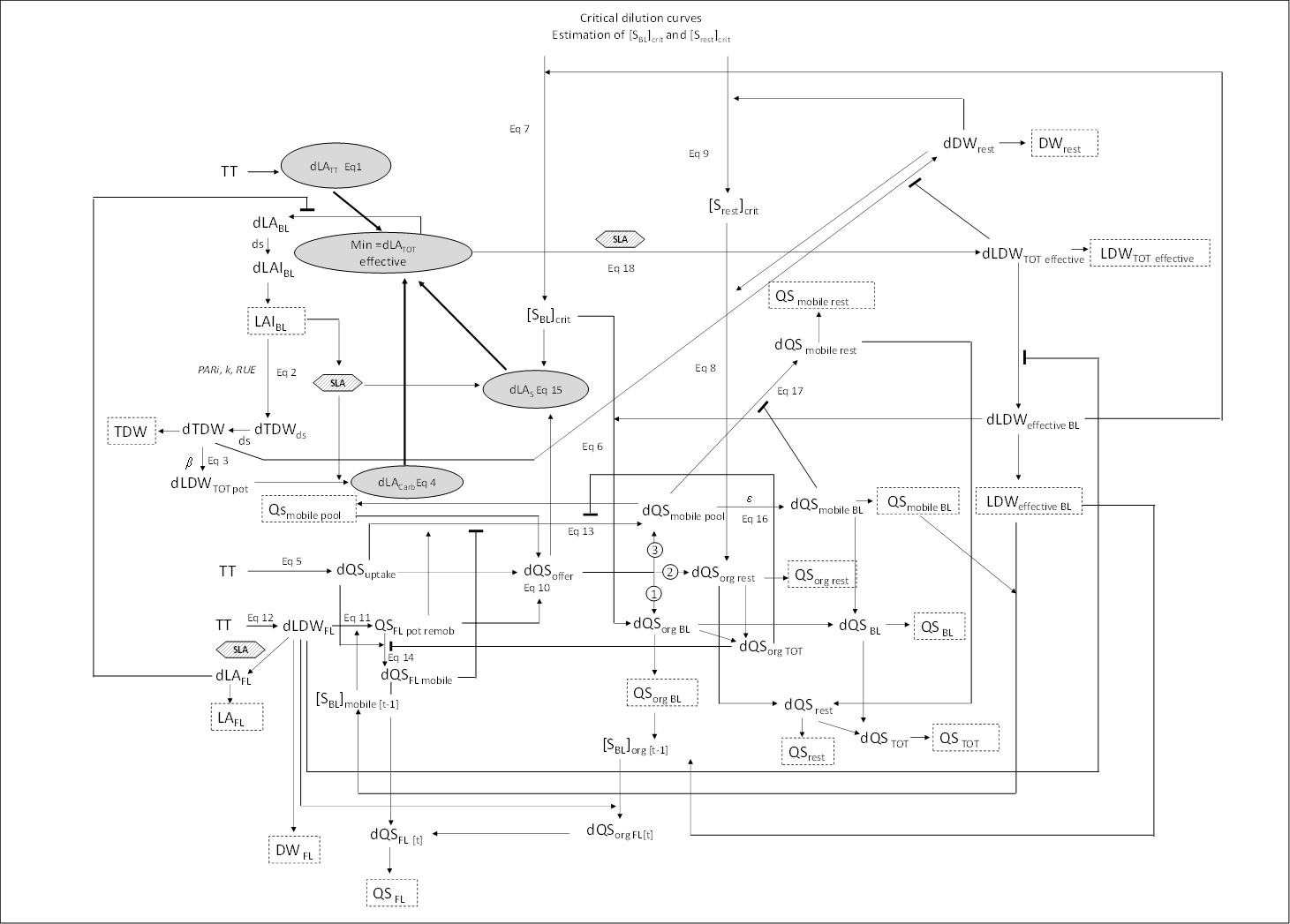

Supplement: Figure S1 — Workflow of SuMoTORI. The model predicts leaf area expansion, biomass and the S amounts in the whole-plant and in the plant compartments and the fraction of organic S (used for growth, i.e., structural and metabolic functions) vs. mobile S (estimated by S from SO42-), for the big leaf, the FL and the rest of the plant (roots, taproot, stem, inflorescences, and pods) from the end of vernalization up to the onset of pod formation. Arrows indicate the precedent and dependent variables. Flat-ended lines represent subtraction of the variable (for details, see the related equation). Numbers in circles indicate the priority order to satisfy S demands from the S offer. State variables are framed with dotted lines. Definitions of the abbreviated variables and parameters are given in Tables 1 and 2. Equations are given in Supplemental Tables S1. [file Image_1.JPEG]
