# Supplementary material for: SuMoToRI, an Ecophysiological Model to Predict Growth and Sulfur Allocation and Partitioning in Oilseed Rape (Brassica napus L.) Until the Onset of Pod Formation
Source: Front Plant Sci. 2015 Nov 17;6:993. doi: 10.3389/fpls.2015.00993 (PMC4647072; doi:10.3389/fpls.2015.00993)
Supplement: TABLE S1 — List of equations. [file Table_1.DOCX]

| **Process and symbol** | **Equation** | **Unit** |
| --- | --- | --- |
|  |  |  |
| **C acquisition and plant offer** | $\text{dTDW}_{\text{ds}}\left[ \text{t} \right]\text{=RUE}\text{ }\text{PAR}_{\text{i}}\left( \text{1-}\text{e}^{\text{-k }\text{LAI}_{\text{BL}}} \right)$  with PAR_abs_= PARi$\left( \text{1-}\text{e}^{\text{-k }\text{LAI}_{\text{BL}}} \right)$ | Eq 2 |
| **C allocation to leaves** | dLDW_TOTpot_ [t] = β dTDW[t] | Eq 3 |
| **Growth S Demand**  α_BL_  β_BL_  α_rest_  β_rest_ | dQS_org BL_ [t] = [S_BL_]_crit_ [t] dLDW _effective BL_ [t]  [S_BL_]_crit_ [t] = α_BL_ LDW_effective BL_^βBL^  dQS _org rest_ [t] = [S_rest_]_crit_ [t] dDW_rest_ [t]  [S_rest_] _crit_ [t] = α_rest_ DW_rest_^βrest^ | Eq 6  Eq 7  Eq 8  Eq 9 |
| **Plant S offer**   1. S uptake 2. S remobilization from fallen leaves 3. S mobile pool | dQS_offer_ [t] = dQS_uptake_[t]+QS_FL pot remob_ [t]+QS_mobile pool_ [t]    QS_FL pot remob_ [t] = dLDW_FL_ [t] [S_BL_] _mobile_ [t-1]  with   dQS_mobile pool_ [t]= {dQS_uptake_ [t]+(QS_FL pot remob_ [t]-dQS_FL_ _mobile_ [t])}-(dQS_org BL_[t]+dQS_org rest_ [t])  with dQS_FL_ _mobile_ [t] = QS_FL pot remob_ [t] if dQS_uptake_ [t]- dQS_org TOT_[t] > 0  or else dQS_FL_ _mobile_ [t] = QS_FL pot remob_ [t] + dQS_uptake_ [t]- dQS_org TOT_[t] | Eq 10  Eq 5  Eq 11  Eq 12  Eq 13  Eq 14 |
| **Leaf area expansion rate according to:**   1. Potential growth 2. C offer and leaf C demand 3. S offer and leaf growth demand |   dLA_carb_ = dLDW_TOTpot_ SLA   | Eq 1  Eq 4  Eq 15 |
| **Mobile S allocation to leaves and to the rest of the plant** | dQS_mobile BL =_ ε(t) dQS_mobile pool_  If dQS_uptake_ [t]-dQS_org TOT_ [t]> 0.2 dQS_uptake_ [t], then ε(t) = ε_opt_  otherwise ε(t) = 0.4 ε_opt_  dQS_mobile rest =_  dQS_mobile pool_ – dQS_mobile BL_ | Eq 16  Eq 17 |
| **Effective leaf area expansion** | dLA_TOT effective_ = min (dLA_TT_, dLA_carb_, dLA_S_)   | Eq 18 |

**Supplemental data Table 1. List of equations**
