# Supplementary material for: SuMoToRI, an Ecophysiological Model to Predict Growth and Sulfur Allocation and Partitioning in Oilseed Rape (Brassica napus L.) Until the Onset of Pod Formation
Source: Front Plant Sci. 2015 Nov 17;6:993. doi: 10.3389/fpls.2015.00993 (PMC4647072; doi:10.3389/fpls.2015.00993)
Supplement: TABLE S2 — Weekly (W) amounts of MgSO4 (mg) and NH4NO3 (mg) per plant for HS and LS treatments. Between W5 and W6, there were 3 weeks of vernalization (Exp1) and 4 weeks of vernalization (Exp2) during which plants of all treatments were provided with a 25% Hoagland nutrient solution (see Material and Methods section). Amounts were calculated from sowing until seed maturity to match physiological stage S-requirements as observed in Dubousset et al. (2010). [file Table_2.DOCX]

|  | MgSO_4_ (mg plant^-1^) | | NH_4_NO_3_ (mg plant^-1^) |
| --- | --- | --- | --- |
|  | HS | LS |  |
| W1 | 55.9 | 0.7 | 0.0 |
| W2 | 42.2 | 0.5 | 0.3 |
| W3 | 71.3 | 0.9 | 1.6 |
| W4 | 109.8 | 1.4 | 6.8 |
| W5 | 156.1 | 2.0 | 23.6 |
| W6 | 218.2 | 2.7 | 74.5 |
| W7 | 280.1 | 3.5 | 146.7 |
| W8 | 325.9 | 4.1 | 230.0 |
| W9 | 363.7 | 4.5 | 325.7 |
| W10 | 391.6 | 4.9 | 409.1 |
| W11 | 408.8 | 5.1 | 480.5 |
| W12 | 415.5 | 5.2 | 535.9 |
| W13 | 412.7 | 5.2 | 554.3 |
| W14 | 401.9 | 5.0 | 406.7 |
| W15 | 384.8 | 4.8 | 381.1 |
| W16 | 363.0 | 4.5 | 345.1 |
| W17 | 338.2 | 4.2 | 310.7 |
| W18 | 311.6 | 3.9 | 278.5 |
| W19 | 284.4 | 3.6 | 241.9 |
| W20 | 257.5 | 3.2 | 210.4 |
| W21 | 231.6 | 2.9 | 183.1 |
| W22 | 207.0 | 2.6 | 155.4 |

**Table 2**: Weekly (W) amounts of MgSO_4_ (mg) and NH_4_NO_3_ (mg) per plant for HS and LS treatments. Between W5 and W6, there were 3 weeks of vernalization (Exp1) and 4 weeks of vernalization (Exp2) during which plants of all treatments were provided with a 25% Hoagland nutrient solution (see Material and Methods section). Amounts were calculated from sowing until seed maturity to match physiological stage S-requirements as observed in Dubousset et al. (2010).
